# High sensitivity for peripartum cardiomyopathy among large language models during differential diagnosis consideration

**DOI:** 10.1177/17455057261476573

**Published:** 2026-08-19

**Authors:** Thomas Gausepohl, Melanie Ricke-Hoch, Denise Hilfiker-Kleiner, Johann Bauersachs, Tobias Jonathan Pfeffer

**Affiliations:** 1 Department of Cardiology and Angiology, 9177 Hannover Medical School, Hannover, Germany; 2 9177 President, Hannover Medical School, Hannover, Germany

**Keywords:** peripartum cardiomyopathy, artificial intelligence, large language model, differential diagnosis

## Abstract

**Background:**

Peripartum cardiomyopathy (PPCM) is a rare, potentially life threatening condition with nonspecific heart failure symptoms, often leading to delayed diagnosis. Large language models (LLMs) may support differential diagnosis suggestion.

**Objectives:**

To assess whether commonly available LLMs can suggest PPCM and differential diagnoses based on typical symptoms.

**Design:**

Comparative evaluation of three LLMs using standardized clinical scenarios.

**Methods:**

ChatGPT (GPT-5), Gemini (2.5 Flash), and Claude (Sonnet 4.5) were queried with prompts describing a 34-year-old woman with PPCM-related symptoms, with and without postpartum context. Responses were analyzed for PPCM and key differential diagnoses recommended by the European Society of Cardiology.

**Results:**

ChatGPT and Claude consistently identified PPCM. Gemini suggested PPCM mainly when dyspnea or edema were present and required postpartum context when palpitations were reported. Claude listed the most differential diagnoses.

**Conclusion:**

LLMs are a valuable tool among differential diagnosis suggestion but performance varies and cannot replace clinical judgment.

## Introduction

When a woman in the peripartum period experiences symptoms such as dyspnea, palpitations, or edema, heart failure and peripartum cardiomyopathy (PPCM) in particular must be considered the potential underlying cause.^1^ PPCM is defined as a deterioration of left ventricular ejection fraction (LVEF) below 45% in the last month of pregnancy, under birth or in the first months after delivery.^1,2^ The incidence of PPCM varies by geographical region and ethnical background: while there exist hotspot regions with incidence rates of up to 1 in 100 live births (parts of Nigeria), in Germany PPCM is a rare condition estimated to occur in 1 in 1,000 to 1,500 live births.^3,4^ The severity of PPCM ranges from mild courses, sometimes even not recognized, to severe forms presenting with cardiogenic shock and the need for mechanical circulatory support.^5^ In the recent years, several clinical and experimental studies have contributed to a better understanding of the pathophysiology and clinical course of the disease, leading to a therapeutic concept, including the use of bromocriptine as a PPCM specific approach that reduces circulating blood prolactin levels in order to inhibit the pathological effects of its PPCM promoting 16-kDa fragment.^6,7^

However, diagnosis of PPCM may be delayed due to the low incidence and therefore low awareness for the disease.^8^

Recently, use and evidence regarding artificial intelligence (AI) including large language models (LLMs) have been growing.^9^ These tools may supplement the list of differential diagnoses considered by the treating physician especially with regard to diseases with low prevalence/awareness such as PPCM.

This study aimed to investigate the sensitivity of three widely used, open access LLMs with regard to PPCM. Furthermore, suggested differential diagnoses of PPCM were compared and analysed with regard to completeness.

## Methods

### Data collection

Each of the three LLMs ChatGPT (GPT-5) (Open AI), Gemini (2.5 Flash) (Google) and Claude (Sonnet 4.5) (Anthropic) using the Web User Interface (Web UI) was requested once to consider differential diagnoses regarding a 34-year-old woman presenting at the emergency department with one or more of the following symptoms: dyspnea, edema, palpitations, dyspnea and edema, dyspnea and palpitations, edema and palpitations, dyspnea and edema and palpitations. Age and symptoms align with those commonly observed in PPCM cases.^3^ Furthermore, the prompt was repeated with the additional information that the woman had given birth two weeks ago. All requests to the LLMs were prompted identically on October 3, 2025 (Table S1).

First, we assessed whether PPCM was explicitly listed mentioned as a differential diagnosis. Second, the differential diagnoses further mentioned were analyzed and compared to those recommended by the Heart Failure Association of the European Society of Cardiology (ESC) Working Group on PPCM.^1^ The naming of the following differential diagnoses was verified if named by the LLM: Myocarditis, pre-existing cardiomyopathy, Takotsubo cardiomyopathy, myocardial infarction, pulmonary embolism, amniotic fluid embolism, hypertensive heart disease/preeclampsia, hypertrophic cardiomyopathy, pre-existing valvular/congenital heart disease, arrhythmia/tachymyopathy.

### Statistical analysis

Data were analyzed using GraphPad Prism version 10.0 for Mac (GraphPad Software, La Jolla California, USA) and are presented as n or median with interquartile range. Test on normal distribution was performed with Shapiro-Wilk test. To compare the number of correct differential diagnoses suggested by three LLMs across the same set of clinical requests, we used the Friedman test.

## Results

### Consideration of PPCM as a differential diagnosis

**Figure 1.**
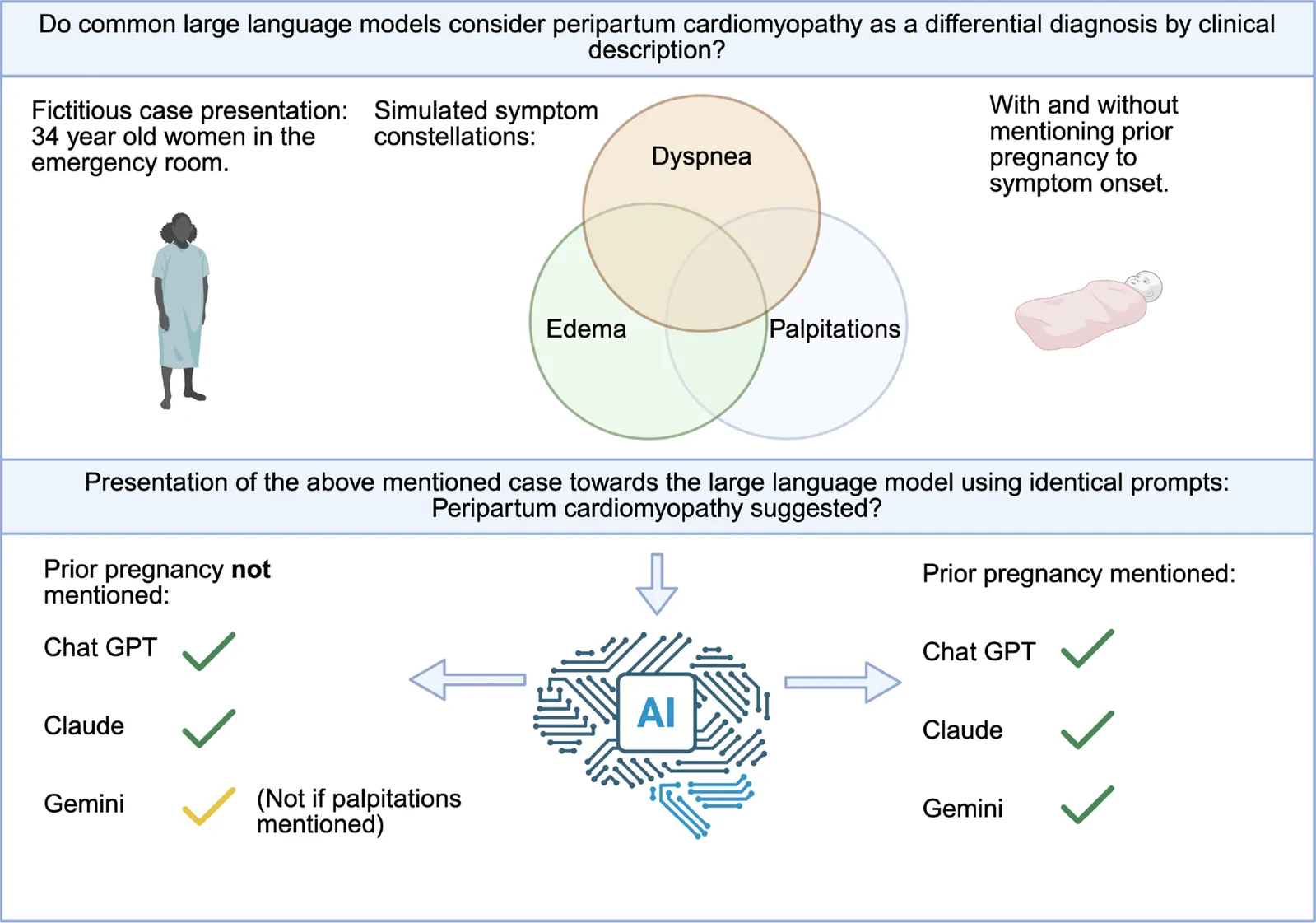
Graphical abstract: Large language model driven differential diagnosis consideration of peripartum cardiomyopathy. The overall study design and key findings are visually summarized in the graphical abstract. Created in BioRender. Pfeffer, T. (2026) https://BioRender.com/7wuaetu.

**Figure 2.**
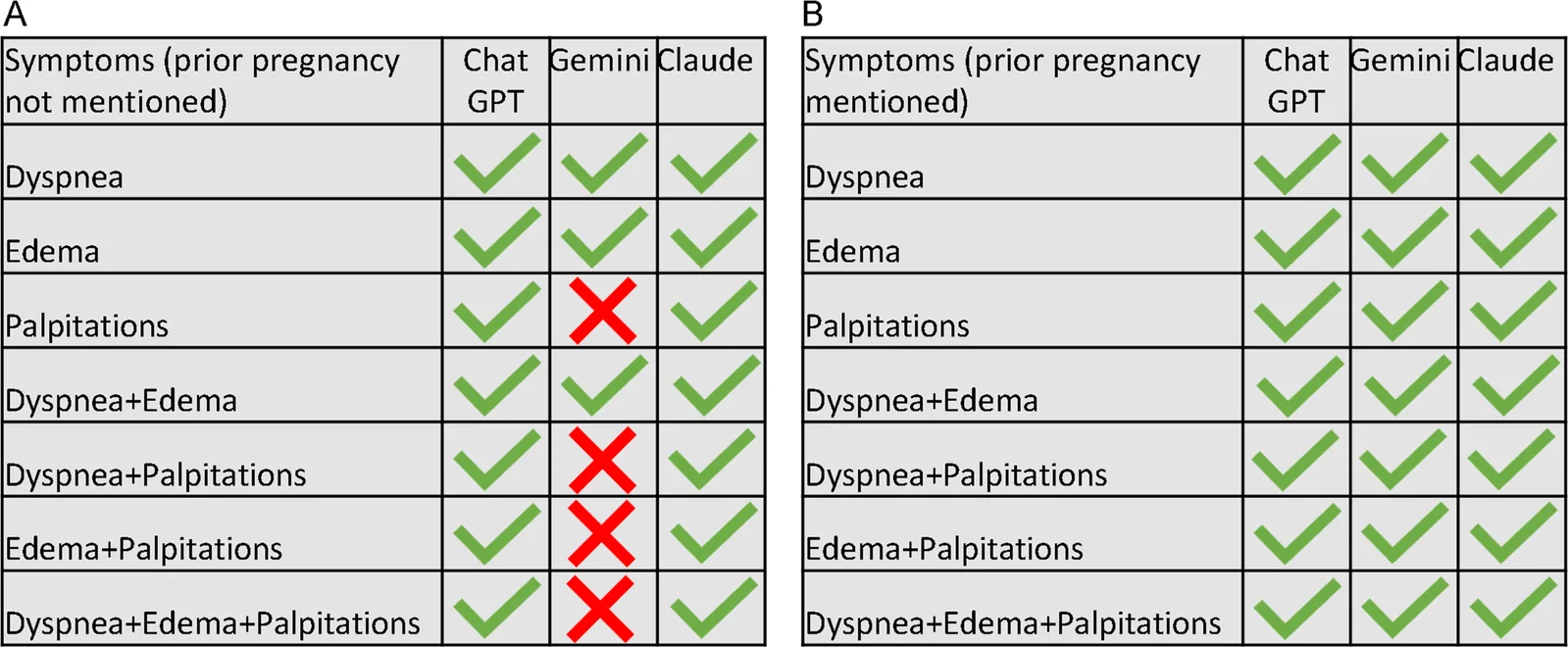
Differential diagnostic consideration of peripartum cardiomyopathy by large language models. The green hook indicates that peripartum cardiomyopathy was listed as a differencial diagnosis, if not the red cross is shown.

Overall, all three LLMs considered PPCM as a differential diagnosis in most cases (Figure 1). When the postpartum status was mentioned, all three LLMs named PPCM as a potential diagnosis in all scenarios. Without the information of the prior pregnancy, ChatGPT and Claude correctly identified PPCM as a differential diagnosis, whereas Gemini failed to do so in several cases (Figure 2).

### PPCM differential diagnoses proposed by ChatGPT, Gemini and Claude

The number of the top 10 PPCM differential diagnoses proposed by ChatGPT, Gemini or Claude is stated in Table 1. If only one symptom was given, dyspnea was the symptom leading to most correct differential diagnoses. In contrast, edema resulted in the lowest relevant number of correct differential diagnoses. If the fictive patient presented with dyspnea and edema, the maximum amount of PPCM differential diagnoses was listed by each LLM. Taken together, Claude showed the most complete PPCM differential diagnosis list stating a median of seven PPCM differential diagnoses, both with and without mentioning the postpartum state of the fictive patient while ChatGPT and Gemini listed four differential diagnoses.

**Table 1.** Range of LLM proposed PPCM differential diagnoses.

Symptoms (n/10)	Prior pregnancy not mentioned				Prior pregnancy mentioned			
	Chat GPT	Gemini	Claude	*p*-value	Chat GPT	Gemini	Claude	*p*-value
Dyspnea	7	6	5	​	5	4	7	​
Edema	3	4	2		3	4	2	
Palpitations	4	4	6		4	4	6	
Dyspnea+Edema	5	7	8		5	7	8	
Dyspnea+Palpitations	5	6	7		5	6	7	
Edema+Palpitations	4	4	7		4	4	7	
Dyspnea+Edema+Palpitations	4	4	8		4	4	8	
Median (Interquartile range)	4 (4-5)	4 (4-6)	7 (5-8)	0.314	4 (4-5)	4 (4-6)	7 (6-8)	0.039*

The amount of the 10 most important PPCM differential diagnoses, which have been listed by the respective large language model (LLM) is stated with regard to the information (symptom/pregnancy history) given to the LLM. Median and interquartile range are stated. *p*-value was estimated with Friedmann test, *=*p* < 0.05.

## Discussion

Delayed diagnosis of PPCM or worsening of pre-existing heart failure during the peripartum period are major drivers of impaired recovery – especially due to the similarity of symptoms.^8,10^ Therefore, raising awareness and enabling early diagnosis and treatment of PPCM is one of the central tasks of PPCM research.^1,11^ After being presented with classic PPCM symptoms, both Chat GPT and Claude considered PPCM as a differential diagnosis, even if the temporal connection to the pregnancy was not mentioned. Gemini showed gaps when palpitations were described, most likely due to fixation error resulting in prioritized suggestion of more common diseases as PPCM like arrhythmias, as these diagnoses are statistically more frequent in training data. This AI “tunnel vision“ - the model’s overreliance on dominant, frequently occurring associations - may impair the ability of LLMs to suggest differential diagnoses, potentially at the expense of rare but critical conditions such as PPCM. The maternal and neonatal outcomes of PPCM are also driven by socioeconomic factors and healthcare resources; thus, the easy access and the free of charge/low cost use of these LLMs may promote diagnosis in low-income regions.^12^ This approach could be supported by AI-based electrocardiography ECG analysis. Lately, Jung et al. showed that AI based analysis of 12-lead ECG was highly effective in screening for PPCM.^13^

In this study, LLMs were evaluated as tools to assist physicians in generating differential diagnoses. As LLM outputs may contain hallucinations, AI-generated suggestions require clinical verification by the treating physician. Importantly, the evidence-based literature necessary for this verification is readily accessible. For example, the ESC Working Group on PPCM position paper is openly accessible and provides standardized, evidence-based recommendations for the diagnosis and management of PPCM.^1^

As PPCM is a diagnosis of exclusion, clarifying differential diagnoses is a central task in the diagnostic work-up of PPCM.^1^ The number of PPCM relevant differential diagnoses suggested by the LLMs varied with the LLM used and the symptoms stated. Claude proved to be the most valuable tool completing this task by providing the most relevant differential diagnoses. Any LLM provided all rule-out diagnoses relevant to PPCM diagnosis, therefore, in case of reasonable suspicion for PPCM resources from the professional associations should be recruited.^1^ Even if LLMs may help in differential diagnosis suggestion, the mentioned resources and clinical judgment must remain central to avoid the risk of over-relying on AI tools and ensure patient-centered decision-making.

### Limitations

While accessing the models via Web UIs accurately mirrors real-world clinical availability, it limited our ability to control backend parameters - such as setting the temperature to zero via an API - to completely eliminate stochastic variance. Furthermore, the clinical scenarios used in this study were simulated: future studies must validate LLM performance in real-world, complex clinical settings to confirm these findings.

LLM based self-diagnosis by medical laypersons with the above mentioned symptoms should not be attempted under any circumstances.

## Conclusions

LLMs like ChatGPT, Gemini, and Claude can support early recognition of PPCM and its differential diagnoses based on symptom input. Claude performed most consistently, while Gemini showed gaps, especially when palpitations were mentioned. LLMs may raise awareness of rare conditions like PPCM, particularly in low-resource settings.

## Supplemental material


Supplemental Material - High sensitivity for peripartum cardiomyopathy among large language models during differential diagnosis consideration
Supplemental Material for High sensitivity for peripartum cardiomyopathy among large language models during differential diagnosis consideration by Thomas Gausepohl, Melanie Ricke-Hoch, Denise Hilfiker-Kleiner, Johann Bauersachs and Tobias Jonathan Pfeffer in Women’s Health.

## Data Availability

The datasets generated and analyzed during the current study are available from the corresponding author on request.*
