# Supplementary material for: High sensitivity for peripartum cardiomyopathy among large language models during differential diagnosis consideration
Source: Womens Health (Lond). 2026 Aug 19;22:17455057261476573. doi: 10.1177/17455057261476573 (PMC13490792; doi:10.1177/17455057261476573)
Supplement: Supplementary file 1 [file sj-pdf-1-whe-10.1177_17455057261476573.pdf]

Supplemental material:

Supplement 1:

Prompting phrases
34-year-old woman presents with dyspnea in the emergency room, please consider the potential differential diagnoses.
34-year-old woman presents with edema in the emergency room, please consider the potential differential diagnoses.
34-year-old woman presents with palpitations in the emergency room, please consider the potential differential diagnoses.
A 34 year old woman presents with dyspnea and edema in the emergency room, please consider the potential differential diagnoses.
34-year-old woman presents with dyspnea and palpitations in the emergency room, please consider the potential differential diagnoses.
34-year-old woman presents with edema and palpitations in the emergency room, please consider the potential differential diagnoses.
34-year-old woman presents with edema and palpitations and dyspnea in the emergency room, please consider the potential differential diagnoses.
34-year-old woman presents with dyspnea in the emergency room two weeks after she gave birth, please consider the potential differential diagnoses.
34-year-old woman presents with edema in the emergency room two weeks after she gave birth, please consider the potential differential diagnoses.
34-year-old woman presents with palpitations in the emergency room two weeks after she gave birth, please consider the potential differential diagnoses.
34-year-old woman presents with dyspnea and edema in the emergency room two weeks after she gave birth, please consider the potential differential diagnoses.
34-year-old woman presents with dyspnea and palpitations in the emergency room two weeks after she gave birth, please consider the potential differential diagnoses.
34-year-old woman presents with edema and palpitations in the emergency room two weeks after she gave birth, please consider the potential differential diagnoses.
34-year-old woman presents with edema and palpitations and dyspnea in the emergency room two weeks after she gave birth, please consider the potential differential diagnoses.

Table S1: Prompts used for LLM evaluation. The used prompts are stated as used for LLM differential diagnosis evaluation.
